# Domestic

**Published:** 1841-02-06

**Authors:** 


### DOMESTIC.

*Death of Dr. Green.*—Jacob Green, M. D., Professor of Chemistry in the Jefferson Medical College, died on Monday, 1st February. Dr. Green was known as an excellent chemist, and an animated, agreeable lecturer. He was much respected, and is generally regretted.

*Albany Medical College.*—The committee on medical colleges of the New York legislature have reported in favour of an appropriation to this institution, for the purchase of a library, and chemical and philosophical apparatus. The present class at this flourishing institution numbers one hundred and twenty-three.

*Treatment of certain Cerebral Symptoms of Scarlatina by Stimulants, instead of Antiphlogistic Remedies.* By T. F. Cornell, M. D.

Dr. Cornell alludes to the error of always regarding the main symptoms of scarlatina as requiring depletion, and sustains what our own experience has long convinced us of, that depletion is in some cases exactly the contrary of the proper remedy. The only difficulty is to distinguish the cases; those given by Dr. C. will probably give a good idea of the points to which he alludes.

I will now subjoin a crude report of two cases of *scarlatina encephalica*, as examples of the condition and treatment for which I have been contending; and for the purpose of farther illustrating my point, I will briefly notice the symptoms and treatment of variola, puerperal fever, and burns, by reporting an instance of each. The two first will be detailed at length, in order that the candid reader may deduce his own conclusions after knowing all the circumstances which controlled them.

*Case* I.—N. C., a boy five years old, was seized on the eve of Sept. 2d, 1840, with fever. He passed a restless night, and was delirious when morning came. He took a dose of senna and salts, which operated three or four times. A faint rash was observed on the chest, abdomen, and loins; the throat was swelled, the skin hot, and the delirium increasing. The day advanced and things grew worse. In the evening of Sept. 3d, I was called to the patient.

Found him delirious; head hot; eyes suffused, and he took but little notice of any thing around him. Pulse 150, but *compressible;* skin warm and dry; rash indistinct; tongue covered with white fur on the middle and posterior parts, while the tip was dry and red; the extremities were of a moderate heat; tonsils were much enlarged, and ash-coloured spots were forming on them.

℞. Tart. antimonii, grs. ij; aq. font. ℥ij; ordered a teaspoonful every fifteen minutes, until emesis was produced; after which, one-twentieth of a grain tart. emet. was given every three hours during the night. Mustard pediluvium, sinapisms to extremities, and barley water for drink.

Sept. 4th, 9 o'clock A. M. The patient had passed a restless night, tossing to and fro on the bed, gritting and gnashing his teeth, screaming out when at all disturbed, and raving furiously. The emetic operated kindly and relieved his throat, and for two hours the delirium abated so much that he talked rationally. His bowels were moved two or three times during the night, but unattended by tenesmus. He is unable to discern objects this morning; the delirium increases; the pupil is unaffected by light; the extremities, skin, tongue, and pulse remain much the same as they were last evening. There appears but little chance for his surviving the day.

℞. Lactis recent. ebul. Oj. Vini Hispan. opt. prep. secund. artem, and give ℥i every hour; one-twentieth gr. of antimony every fonr hours. Pediluvium capsici, and frictions with infus. capsici on the trunk and extremities; ammoniacal linim. to the throat. He was so unmanageable as to defy our using any application to the tonsils.

4 P. M. Symptoms were decidedly worse; the rash was somewhat more distinct, but he acted like a maniac. The features of the case were truly alarming; venesection was proposed as a dernier resort, although it militiated against the theory I entertained, as to the cause of his furious delirium. But as it corresponded with the practice instituted for like symptoms in other cases, and as it was more in accordance with the old school views, three ounces of blood were drawn from the arm. The effect on the pulse was perceptible ere the ligature was removed; the delirium abated for a few minutes only, and then returned; the eruption became less distinct; the extremities grew cold, and a livid hue overspread the whole surface. I felt alarmed at my proceedings, saw my error and was convinced. Stimulants energetically administered would be the only possible method of saving the patient. *Wine whey* was accordingly given every half hour in as large quantities as he would take, which averaged about ℥i at each time. The antimonial was discontinued, frictions with capsicum and flying sinapisms, were attentively persevered in, until reaction should again be established.

10 P. M. The patient lies comatose; mouth open; grits his teeth when disturbed, and screams out; pupils insensible; refuses to take much drink; continued whey and frictions.

Sept. 5th, 9 A. M. He passed a bad night; the nurse was fearful he would not live till morning. He drank but little, and that was one gill of wine whey; notices nothing; groans out and gnashes his teeth. The tongue is moister than it has been during his attack; skin less hot; eruption more full on body and extremities; pulse 140; pupils acted on by strong light; bowels confined.

℞. Sulph. magnes. ʒi; wine whey and barley water. To keep the room quiet and dark.

9 P. M. Has rested quite well during the day; bowels opened once; drank freely of the whey; eruption complete; tongue moist; throat less tumid; ulcers on the tonsils. He put out his tongue for me when requested; is conscious for a few moments only, and then sinks into a somnolent state. Continued the whey.

Sept. 6th, 9 A. M. Appears decidedly improved. He knew me for the first time since my attendance; is rational; his delirium has subsided; throat bad; deglutition difficult; pulse, tongue, and countenance more natural.

Nit. argenti, grs. vi: aq. dist. ℥i. to pencil the tonsils with it; whey, &c. as before.

8 P. M. Continues to convalesce; has been free from delirium during the day. He has taken three gills of the best Madeira wine made into whey during the last 48 hours, and has had one evacuation from the bowels. To continue the treatment of the morning.

Sep. 7th, 9 A. M. Improvement is progressive; throat is better; left tonsil sloughing; urine free, eruption fading; pulse 140 and soft; tongue moist over its entire surface; skin of natural heat; prostration great.

℞. Sulph. quininæ, grs. iv. arom. sulph. acid gtt. vi. syr. zingiberis, aq. fort. *aa* ℥ss. M. ʒi. every four hours. This boy continued on the quinine and wine whey for several days, and was playing about the room in two weeks from his first attack, and he had no relapse, nor any of the sequelæ incident to scarlatina. Mild but nutritious diet, with an occasional laxative was enjoined, and I ceased to visit him.

*Case* II.—A. D. a girl aged 4 years, was seized with fever on the evening of June 27, 1840, which was attended with vomiting and purging. The child remained in this state until morning, when it became unconscious of all around her. The vomiting now ceased but the diarrhœa continued.

I was called to the case on the 28th, at 8 o'clock, A. M. I found the girl insensible, head hot, and reclined back; mouth open, eyes rolled up and semi-closed. In a short time, she screamed out and uttered unconnected sentences, tossed about the bed, and understood nothing that was said to her. Her tongue was red and dry, with a white fur here any there; respiration quick and laboured, pulse 145, and *compressible;* abdomen was tumid, and tender to the touch; extremities cold, eruption very faint, except on the spine and chest. Discharges from bowels were green, slimy, and fetid.

℞. Misturæ cretæ ℥ij. Tinct. Cardam. Comp. ʒi., Tinct. Opii ʒss. to be given after each stool; vessicat. lyttæ, to the nape of the neck. Cloths wet with cold water to be applied to the head. Sinapisms to the abdomen and the extremities, wine whey and arrowroot with brandy as drink. Starch enemata with Tinct. Opii to arrest diarrhœa.

6 o'clock, P. M. Delirium has increased, heat of head continues, pulse fuller and not so frequent, diarrhœ less urgent, the discharges are passed *involuntarily*, the extremities are warmer. Has drank a gill of wine whey, and the same quantity of arrowroot, with half oz. of brandy in it. Continued the same treatment.

29th, 9 o'clock, A. M. Passed a poor night, knows no one; is very unmanageable when touched or spoken to, frequently screams out and then falls into a sleep. Continued the whey, chalk mixture, arrowroot, and brandy.

6 o'clock, P. M. She is more composed, and drinks the whey freely; still remains unconscious of anything. Has slept quietly at times during the day; eruption remains indistinct. Throat is not very sore, tongue moist; blister discharges very free from neck; pressure on abdomen excites uneasiness, diarrhœa checked. Continued same prescription.

30th, 9 o'clock, A. M. Passed last night better than any since attack; is rational at times this morning, talks some. Pulse 130, compressible.

6 P. M. Improvement is manifestly great. Continued whey, brandy, &c.

July 1, 9 A. M. There no longer remains any doubt that the patient is decidedly better; she is entirely rational this morning. Rash is out full and complete; uniform warmth of the body, tongue moist. No passage from bowels for last twenty-four hours. Prescription of wine whey, arrowroot, brandy and chicken broth.

2d, 9 o'clock, A. M. Is convalescing finely. Asks for her drink, and takes considerable notice of things, for the first time since her sickness; complains of the blister. Prescription, Ol. Ricini ʒiss.; wine whey, arrowroot, chicken broth, soda biscuit, &c.

As no untoward symptoms occurred during her convalescence, I will not detain the reader by a daily report. On the eighth day, the girl was sitting up, and complained only of debility, which a few days' improvement counteracted. The quantity of wine used was one pint in five days, besides one gill of brandy with the arrowroot. The child has passed the summer in good health.

I will now add the cases of burns and variola, because their treatment was on the principle laid down in this essay, and they will assist me in elucidating the subject more fully.

*Case* I.—A girl, 5 years old, had been extensively burned by her frock taking fire. She had a convulsion on the third day, which lasted four hours, and was attended with a hot skin and suffused face. The pulse was *frequent* and *soft*, extremities rather cool. The tongue was precisely like that of scarlatina, where the fur would peal off and leave it morbidly red, with the papillæ elevated. Such was her condition when I was called in. I ordered wine whey immediately, and put her under the use of quinine, ½ gr. every four hours. The convulsions ceased after three hours, but delirium continued during the evening and night. On the following day the cerebral symptoms had subsided, and the febrile condition was removed. The case continued on the use of quinine, with stimulants, and nourishing diet, for several days, and the burns healed up rapidly.

*Case* II.—A boy four years old, was extensively scalded. On the second day he was seized with apparently high fever, accompanied with delirium and convulsions. The tongue was hot and dry, papillæ elevated and red. Pulse *frequent* and *soft*, abdomen tumid, diarrhœa profuse, feet cool. He was treated with brandy toddy, and arrowroot, chalk mixture, and Dover powders, with starch enemas, and then put on quinine. In a few days all the cerebral difficulty had subsided. Profuse suppuration resulted from the deep injury of the scald. A repetition of the quinine was decidedly serviceable in checking it, and causing cicatrization, and the case progressively improved.

*Variola*—in a girl aged nine years.—On the evening of October 10th, 1840, I was called to the patient, and was informed she had been vomiting every thing she had taken during the day; she had been purged four or five times by a dose of senna and manna given the day previous. She now complained of intolerable headach, and was delirious the greater part of the time. The face was suffused, the skin of natural warmth, tongue small and coated, pulse soft and frequent, feet cool. ℞. Spts. Mindereri ʒi. every two hours. Sinapisms to epigastrium and feet. Cold toast-water in small quantities for drink.

11th, 9 A. M. Vomiting had ceased. She still complains of great headach, and is delirious; tosses about the bed; is very restless and moans much of the time. When examining the tongue, I observed a few small *vesicles* on the under lip, which inclined me to think some eruption might be making its appearance; and the arm-pits and groins I found evinced some minute livid points, resembling in size and appearance gunpowder grains. Variola was at once, and for the first time, now suspected. Wine whey was immediately ordered, (one part of wine to two parts of milk,) of which one ounce was to be given every hour; and arrowroot as common drink.

4, P. M. Eruption continues the same in appearance, and is becoming more extensive over the body. Cerebral symptoms no worse; pulse weak; no diarrhœa nor vomiting.

℞. Sulph. Quiniæ, gr. xij.; Arom. Sulph. Acid, gtt. xv.; Syr. Zingiberis, ℥i.; Cap. ʒi. every two hours. Continue wine whey and wine in the arrowroot, as freely as she will drink it.

12th, 9 o'clock, A. M. Eruption still continues livid, and has extended from the body over the extremities. She passed an uneasy night, and was delirious. Bowels confined since the 10th, but no evacuation is desirable. Pulse soft; skin more uniformly warm. Headach somewhat better this morning; tongue cleaning off.

Continue wine whey and quinine in the same dose and interval—chicken broth.

13th, 9 o'clock, A. M. Eruption less livid. Pulse more vigorous, and the general exhaustion not so distressing. No symptoms were present which indicated a change of treatment, and it was continued with the addition of warm gin and water; for she had become tired of whey.

14th. Symptoms are encouraging. She had drank wine in arrowroot; chicken broth and gin and water; together with the quinine every two hours.

The eruption is filling out, and distinct pox are forming of a natural appearance. The bowels have not been moved since the tenth, nor have any laxatives been given since I was called to the case. The *pulse* forbade evacuants, although the encephalon might have demanded local relief.

15th, 9 o'clock, A. M. The patient continues to do well.

The bowels were spontaneously moved once this morning.

17th. All is going on well. The head is as easy as could be expected, and is somewhat swelled. Eyes are closed; pulse soft, 100; tongue clean; temperature natural; eruption confluent, and pus is becoming visible in the pox. The bowels were spontaneously moved to-day.

Up to this time (including six days) the child has taken arrowroot, chicken broth and barley water as drinks; together with one pint and a half of wine, either made into whey, or used with arrowroot. Also, five gills of gin made into sling; and has taken forty-five grains of quinine; while laxatives, relaxants and evacuants have been most carefully avoided. The case continued improving, and was entirely restored to health before the month was past.

*Puerperal Fever.*—Oct. 9th, 1840. Mrs. E., ætat. 32, had been confined with her third child. She had an easy labour of three hours, and no unfavourable symptom presented itself during the first week. On the ninth day after her delivery, she was suddenly seized with rigors which almost amounted to an ague, and were soon followed by flushings of heat, great pain in the head, abdomen and loins. The lochiæ were suppressed, and the secretion of milk much diminished. The pain continued for some hours unabated, and the febrile action was becoming more violent, when I was summoned to visit the lady.

9 o'clock, A. M. I found the patient as above described. Her pulse was 140, but weak; the skin hot and dry, and the pain intolerable—to use her own expression, she ached all over. A pediluvium was ordered instanter; a fomentation of hops and whiskey across the abdomen, and Ol. Ricini ℥i. These remedies gave transient relief, but the pain returned in a short time, and the fever continued. Having resolved to try opiates in large doses when a case approximating to puerperal fever should next present itself to me, I thought this one was sufficiently decided, and its symptoms required prompt alleviation. Having evacuated the bowels, two of the following pills were ordered every hour and a half until the pain should cease.

℞. Pulv. Opii. grs. ix.; Pulv. G. Camphoræ, grs. xxiv.; Pulv. Ipecac. grs. v.; Sulph. Quiniæ, grs. viij.; Muc. G. Acaciæ, q. s. ft. Pil. xij.

6 o'clock, P. M. Nine pills were given before any relief was obtained. The skin is now moist; the pulse less frequent; the pain in the head, abdomen and loins had greatly subsided. The remaining pills were to be given during the night, if the symptoms required them.

10th, 9 A. M. Passed a good night, but the pain returned in the morning—now, she cannot bear the least pressure on her abdomen. The milk remains much diminished, but there is a slight return of the lochiæ this morning. The pulse is frequent and weak, the tongue furred, and the skin moist. There is no severe pain in the head. Barley water and arrowroot were used as drink. The fomentation and pediluvium are to be repeated, and two of the following pills taken every four hours:

℞. Pulv. Opii. grs. vi.; P. Camphoræ, grs. xx.; Sulph. Quiniæ, grs. xij.; ft. Pill, xij.

8 o'clock, P. M. The nurse says, the patient is now entirely free from pain, and has fallen asleep; there is a gentle perspiration over the entire surface, and I left her undisturbed to be refreshed by repose.

11th, 10 o'clock, A. M. Rested well during the night; is free from pain and fever this morning, but complains of being very sore. Lochiæ and milk abundant. A laxative was now prescribed; light diet, and Sulph. Quiniæ, ℈i.; Elix. Vit. gtt. xxx.; Syr. Zingiberis ℥ij. M. To take a teaspoonful every six hours. The object of the quinine was to fortify the system, and thereby prevent a return of the pain and fever. In this it succeeded; for she was soon enabled to leave her room with impunity, and during the ensuing month had no relapse.

The above cases are not reported with that nicety which my inclination would have prompted; but, as they were noted in much haste, and at a time when I had no thought of laying them before the public, I trust the execution will not effect the reader's deductions. In remarking on the general treatment adopted in the cases of scarlatina and burns, I have only to say, that formerly I attempted to relieve the brain symptoms by V. S.; leeches, warm bath, purgatives, enemas, relaxants, and counter-irritants; and three-fourths thus treated died. I reasoned that there had been a transfer or metastasis of irritation to the brain, or that incipient encephalitis was the fruitful source of so many formidable symptoms. My practice was of course energetic, and the effect speedy, but unsatisfactory. As to the case of puerperal fever, some might question it being genuine—it was such, however, as to satisfy my own mind. At all events, I have been informed by intelligent members of the profession, that these were the symptons for which a score of leeches over the abdomen, with ice and cups to the head, drastics and blisters, &c., would be continually used, and *their* cases would die of "puerperal fever!" I am aware it is difficult to reconcile long cherished principles and established practices, with opposite sentiments and different treatment; but, venerable as any sanctioned course may be rendered by time, and plausible as it may seem to a thoughtless observer, yet well attested facts and correct observations are the only true methods of obtaining practical deductions. To clinical observations, I would refer any who are curious on the subject of this Essay; and if the views which it advances should prove hollow and feeble, let them pass into merited oblivion. But, if after a fair and thorough examination, clinical results should demonstrate the correctness of its principles, let them be diffused for the welfare of humanity and the promotion of science.—*New York Journal of Medicine and Surgery.*

---

*Medical Faculty of the University of the City of New York.*—The following appointments have just been made in this institution:—Hon. Theodore Frelinghuysen, Chancellor of the University, President of the Faculty. Granville Sharp Pattison, M. D., Professor of General, Descriptive, and Surgical Anatomy. Valentine Mott, M. D., Professor of the Principles, Practice and Operations of Surgery. John Revere, M. D., Professor of the Theory and Practice of Medicine. Martyn Paine, M. D., Professor of the Institutes of Medicine and Materia Medica. Gunning S. Bedford, M. D., Professor of Midwifery and the Diseases of Women and Children. John Williams Draper, M. D., Professor of Chemistry.

INTERMENTS *in the City and Liberties of Philadelphia, from the 2nd to the 30th of January,* 1841.

| Diseases. | Adults. | Children. |
|---|---|---|
| Abscess, | 2 | 0 |
| —— of the brain, | 1 | 0 |
| —— of the liver, | 1 | 0 |
| —— lumbar, | 1 | 0 |
| Angina pectoris, | 1 | 0 |
| Apoplexy, | 4 | 0 |
| Asphyxia, | 0 | 1 |
| Burns, | 0 | 2 |
| Casualties, | 4 | 0 |
| Childbed, | 1 | 0 |
| Compression of the brain, | 0 | 1 |
| Congestion of the lungs, | 1 | 1 |
| Consumption of the lungs, | 39 | 8 |
| Convulsions, | 3 | 19 |
| —— Puerperal, | 1 | 0 |
| Croup, | 0 | 4 |
| Debility, | 1 | 5 |
| Diarrhœa, | 2 | 1 |
| Disease of the brain, | 0 | 1 |
| —— head, | 0 | 1 |
| —— heart, | 3 | 4 |
| —— liver, | 1 | 0 |
| —— lungs, | 0 | 2 |
| —— stomach, | 1 | 0 |
| Dropsy, | 2 | 0 |
| —— abdominal, | 2 | 0 |
| —— breast, | 2 | 1 |
| —— head, | 0 | 8 |
| —— ovarian, | 1 | 0 |
| Drowned, | 1 | 0 |
| Dysentery, | 1 | 0 |
| Empyema, | 0 | 1 |
| Enlargement of the heart, | 2 | 0 |
| Executed, | 1 | 0 |
| Exposure to cold, | 1 | 0 |
| Fever, | 0 | 1 |
| —— bilious, | 1 | 0 |
| —— catarrhal, | 0 | 4 |
| —— puerperal, | 1 | 0 |
| —— scarlet, | 0 | 10 |
| —— typhoid, | 1 | 0 |
| —— typhus, | 6 | 1 |
| Gangrene, | 1 | 1 |
| Hernia, | 0 | 1 |
| Hooping-cough, | 0 | 2 |
| Inflammation of the brain, | 7 | 7 |
| —— breast, | 0 | 3 |
| —— bowels, | 1 | 1 |
| *Carried forward,* | 98 | 91 |
| *Brought forward,* | 98 | 91 |
| Infl. of bronchi, | 1 | 5 |
| —— larynx, | 0 | 1 |
| —— liver, | 1 | 0 |
| —— lungs, | 5 | 6 |
| —— peritoneum, | 1 | 0 |
| —— stomach, | 2 | 1 |
| —— stomach and bowels, | 1 | 0 |
| Insanity, | 1 | 0 |
| Intemperance, | 2 | 0 |
| Jaundice, | 0 | 1 |
| Laudanum to excess, | 1 | 1 |
| Mania a potu, | 1 | 0 |
| Marasmus, | 2 | 4 |
| Measles, | 0 | 1 |
| Morbus cœruleus, | 0 | 1 |
| Old age, | 9 | 0 |
| Palsy, | 3 | 0 |
| Rupture of a blood vessel, | 1 | 0 |
| Scirrhus of the uterus, | 1 | 0 |
| Small pox, | 2 | 7 |
| Spitting of blood, | 1 | 0 |
| Still-born, | 0 | 40 |
| Suffocation, | 1 | 0 |
| Tabes mesenterica, | 0 | 1 |
| Teething, | 0 | 1 |
| Tumours, | 0 | 1 |
| Ulceration of larynx, | 1 | 1 |
| —— bowels, | 1 | 0 |
| Unknown, | 1 | 4 |
| Total, | 137 | 167 |

Of the above, there were under 1 year, 105

| | | |
|---|---|---|
| From | 1 to 2 | 25 |
| | 2 to 5 | 21 |
| | 5 to 10 | 4 |
| | 10 to 15 | 4 |
| | 15 to 20 | 9 |
| | 20 to 30 | 33 |
| | 30 to 40 | 27 |
| | 40 to 50 | 18 |
| | 50 to 60 | 22 |
| | 60 to 70 | 11 |
| | 70 to 80 | 16 |
| | 80 to 90 | 7 |
| | 90 to 100 | 2 |
| Total, | | 304 |

In the above are included thirty-eight people of colour, and five interments from the country.

